# 1-(4-Bromo­phen­yl)-1-(4-nitro­benzo­yl)thio­urea

**DOI:** 10.1107/S1600536809025884

**Published:** 2009-07-15

**Authors:** Sohail Saeed, Naghmana Rashid, Arifa Tahir, Peter G. Jones

**Affiliations:** a Department of Chemistry, Research Complex, Allama Iqbal Open University, Islamabad, Pakistan; b Environmental Science Department, Lahore College for Women University, Lahore 54000, Pakistan; c Institut für Anorganische und Analytische Chemie, Technische Universität Braunschweig, Postfach 3329, 38023 Braunschweig, Germany

## Abstract

The title compound, C_14_H_10_BrN_3_O_3_S, crystallizes as two concomitant polymorphs that differ in colour (one yellow and one colourless). Only the structure of the colourless form could be determined. The mol­ecule exists in the thio­amide form with an intra­molecular N—H⋯O=C hydrogen bond across the thio­urea system. Mol­ecules are linked into layers parallel to (120) by Br⋯O_nitro_ contacts [3.103 (1) Å], classical hydrogen bonds from the other NH function to the S atom and N_nitro_⋯O=C contacts. The layers are linked by weak C—H⋯O_nitro_ hydrogen bonds to produce the observed three-dimensional network.

## Related literature

For general background to thio­urea complexe, see: Ugur *et al.* (2006 ▶). For the biological activity of thio­urea derivatives, see: Glasser & Doughty (1964 ▶); Huebner *et al.* (1953 ▶); Manjula *et al.* (2009 ▶); Zheng *et al.* (2004 ▶). For related structures, see: Saeed *et al.* (2008*a* ▶,*b* ▶,*c* ▶). 
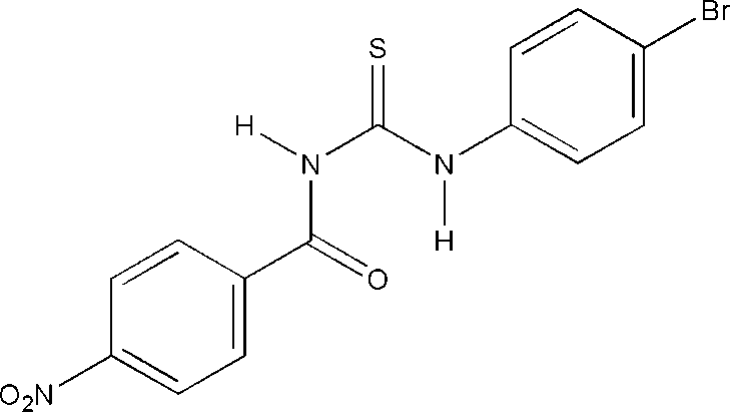


## Experimental

### 

#### Crystal data

C_14_H_10_BrN_3_O_3_S
*M* *_r_* = 380.22
Triclinic, 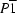

*a* = 7.0112 (3) Å
*b* = 8.9697 (5) Å
*c* = 11.9693 (6) Å
α = 87.386 (5)°
β = 75.044 (4)°
γ = 87.511 (4)°
*V* = 726.08 (6) Å^3^
*Z* = 2
Mo *K*α radiation
μ = 2.99 mm^−1^
*T* = 100 K
0.3 × 0.2 × 0.2 mm

#### Data collection

Oxford Diffraction Xcalibur Eos diffractometer
Absorption correction: multi-scan (CrysAlis Pro; Oxford Diffraction, 2009 ▶) *T*_min_ = 0.945, *T*_max_ = 1.000 (expected range = 0.520–0.550)
24890 measured reflections
4234 independent reflections
3438 reflections with *I* > 2σ(*I*)
*R*_int_ = 0.025

#### Refinement

*R*[*F* ^2^ > 2σ(*F* ^2^)] = 0.022
*wR*(*F* ^2^) = 0.051
*S* = 0.96
4234 reflections
207 parameters
2 restraints
H atoms treated by a mixture of independent and constrained refinement
Δρ_max_ = 0.81 e Å^−3^
Δρ_min_ = −0.80 e Å^−3^

### 

Data collection: *CrysAlis Pro* (Oxford Diffraction, 2009 ▶); cell refinement: *CrysAlis Pro*; data reduction: *CrysAlis Pro*; program(s) used to solve structure: *SHELXS97* (Sheldrick, 2008 ▶); program(s) used to refine structure: *SHELXL97* (Sheldrick, 2008 ▶); molecular graphics: *XP* (Siemens, 1994 ▶); software used to prepare material for publication: *SHELXL97*.

## Supplementary Material

Crystal structure: contains datablocks I, global. DOI: 10.1107/S1600536809025884/im2125sup1.cif

Structure factors: contains datablocks I. DOI: 10.1107/S1600536809025884/im2125Isup2.hkl

Additional supplementary materials:  crystallographic information; 3D view; checkCIF report

## Figures and Tables

**Table 1:** Hydrogen-bond geometry (Å, °)

*D*—H⋯*A*	*D*—H	H⋯*A*	*D*⋯*A*	*D*—H⋯*A*
N1—H01⋯O1	0.82 (1)	2.03 (2)	2.688 (2)	138 (2)
N2—H02⋯S^i^	0.77 (1)	2.79 (1)	3.553 (1)	169 (1)
C5—H5⋯O3^ii^	0.95	2.38	3.293 (2)	162
C3—H3⋯S^iii^	0.95	2.93	3.474 (2)	118
C14—H14⋯S^i^	0.95	2.89	3.167 (1)	98
C14—H14⋯Br^iv^	0.95	3.15	3.899 (1)	137

Symmetry codes: (i) 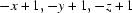; (ii) 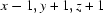; (iii) 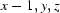; (iv) 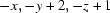.

**Fig. 1.**
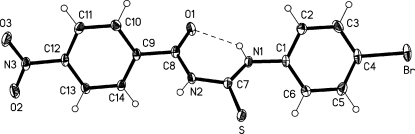
The molecular structure of the title compound. Ellipsoids correspond to 50% probability levels.

**Fig. 2.**
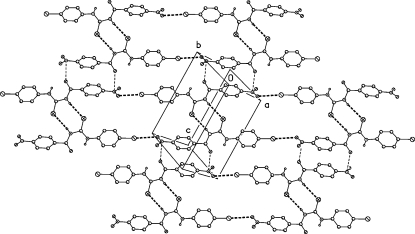
Packing diagram of the title compound, showing classical hydrogen bonds and Br···O contacts (thick dashed lines) and N···O contacts (thin dashed lines). View direction: perpendicular to (120).

## supplementary crystallographic information

### Comment

Industrial production and the use of elements such as Fe, Co, Cu, Ni, Zn, Cd and Pb can cause environmental pollution. However, some of these metals are present in trace amounts as essential elements for biological systems and also play an important role in bioinorganic chemistry. In order to understand the role of these metal ions in biological systems, structural studies of biological compounds and their metal complexes are extremely important. Compounds containing carbonyl and thiocarbonyl groups occupy an important position among organic reagents as potential donor ligands for transition metal ions (Ugur et al., 2006).

Thioureas are also known to exhibit a wide range of biological effects including antiviral, antibacterial, anticancer (Manjula et al., 2009), antifungal, antitubercular, antithyroidal, herbicidal and insecticidal activities (Huebner et al., 1953) and are used as agrochemicals (Saeed et al., 2008a<i/>). An example is furnished by 1-benzoyl-3-(4,5-disubstituted-pyrimidine-2-yl)- thioureas that have excellent herbicidal activity (Zheng et al., 2004). Thioureas are also well known chelating agents for transition metals (Saeed et al., 2008b<i/>). The complexes of thiourea derivatives also show varied biological activities (Glasser et al., 1964). Thioureas and substituted thioureas are also known as epoxy resin curing agents (Saeed et al., 2008b<i/>). We became interested in the synthesis of N-aroyl, N'-arylthioureas as intermediates towards some new heterocyclic compounds and for the systematic study of their bioactive complexes and their function as epoxy resin curing agents, and have recently published three structures from this compound class (Saeed et al., 2008a,b,c).

The molecule of the title compound is shown in Fig. 1. It crystallizes in the thioamide form with an intramolecular hydrogen bond N1—H01···O1. Bond lengths and angles may be regarded as normal. The central moiety N1—C7( S)—N2—C8(O1) is essentially planar (mean deviation 0.09 Å) and subtends interplanar angles of 38.42 (4)° and 56.27 (4)° with the aromatic rings at C1 and C9, respectively. This is also reflected by the corresponding torsion angles C7—N1—C1—C6 43.7 (2)° and N2—C8—C9—C14 - 50.8 (2)°.

The molecular packing is determined by a variety of intermolecular contacts. The molecules are linked to chains parallel to [211] by the halogen bond Br···O2 3.103 (1) Å, with an angle C4—Br···O2 of 176.07 (5)° (operator: x - 2, y + 1, z + 1). The chains are crosslinked by the classical H bond N2—H02···S (Table 1) and the contact N3···O1 2.957 (1) Å, with an angle C12—N3···O1 of 88.60 (7)° (operator: -x + 1, -y + 1, -z), to form layers parallel to (120). The layers are connected by the weak H bond C5—H5···O3 to give the observed three dimensionial structure (Table 1). Further borderline H···X interactions are also listed in Table 1 for the sake of completeness but are probably of minimal structural relevance.

### Experimental

A solution of 4-nitrobenzoyl chloride (0.1 mol) in dry acetone (80 ml) was added dropwise to a suspension of ammonium thiocyanate (0.1 mol) in acetone (50 ml) and the reaction mixture was refluxed for 30 minutes. After cooling to room temperature, a solution of 4-bromoaniline (0.1 mol) in acetone (25 ml) was added and the resulting mixture refluxed for 1.5 h. The reaction mixture was poured into five times its volume of cold water, upon which the thiourea precipitated. The product was recrystallized from ethyl acetate as intensely yellow crystals. However, these crystals, despite near-perfect optical appearance under the microscope, proved to be unusable for X-ray analysis because their diffraction patterns were very weak and diffuse. A few large colourless prisms were therefore selected from the same sample and proved to be of diffraction quality. Clearly the title compound is polymorphic, and one may speculate that the different colours may arise from different H bonding patterns.

### Refinement

The NH hydrogen atoms were refined freely, but with an N—H distance restraint of 0.82 Å and an associated notional e.s.d. of 0.02 Å (command *DFIX*). Other H atoms were placed in calculated positions and refined using a riding model with C—H 0.95 Å; the hydrogen *U* values were fixed at 1.2 × *U*(eq) of the parent atom. The largest features of residual electron density (*ca* 0.8 e Å^-3^) lie within 1 Å of the Br atom.

### Crystal data

**Table:**

C_14_H_10_BrN_3_O_3_S	*Z* = 2
*M**_r_* = 380.22	*F*(000) = 380
Triclinic, *P*1	*D*_x_ = 1.739 Mg m^−^^3^
Hall symbol: -P 1	Melting point: 454 K
*a* = 7.0112 (3) Å	Mo *K*α radiation, λ = 0.71073 Å
*b* = 8.9697 (5) Å	Cell parameters from 13461 reflections
*c* = 11.9693 (6) Å	θ = 2.3–31.7°
α = 87.386 (5)°	µ = 2.99 mm^−^^1^
β = 75.044 (4)°	*T* = 100 K
γ = 87.511 (4)°	Block, colourless
*V* = 726.08 (6) Å^3^	0.3 × 0.2 × 0.2 mm

### Data collection

**Table:**

Oxford Diffraction Xcalibur Eos diffractometer	4234 independent reflections
Radiation source: Enhance (Mo) X-ray Source	3438 reflections with *I* > 2σ(*I*)
graphite	*R*_int_ = 0.025
Detector resolution: 16.1419 pixels mm^-1^	θ_max_ = 30.0°, θ_min_ = 2.3°
ω scans	*h* = −9→9
Absorption correction: multi-scan (*CrysAlis PRO*; Oxford Diffraction, 2009)	*k* = −12→12
*T*_min_ = 0.945, *T*_max_ = 1.000	*l* = −16→16
24890 measured reflections	

### Refinement

**Table:**

Refinement on *F*^2^	Primary atom site location: structure-invariant direct methods
Least-squares matrix: full	Secondary atom site location: difference Fourier map
*R*[*F*^2^ > 2σ(*F*^2^)] = 0.022	Hydrogen site location: inferred from neighbouring sites
*wR*(*F*^2^) = 0.051	H atoms treated by a mixture of independent and constrained refinement
*S* = 0.96	*w* = 1/[σ^2^(*F*_o_^2^) + (0.0305*P*)^2^] where *P* = (*F*_o_^2^ + 2*F*_c_^2^)/3
4234 reflections	(Δ/σ)_max_ = 0.002
207 parameters	Δρ_max_ = 0.81 e Å^−^^3^
2 restraints	Δρ_min_ = −0.79 e Å^−^^3^

### Special details

**Table:**

Geometry. All e.s.d.'s (except the e.s.d. in the dihedral angle between two l.s. planes) are estimated using the full covariance matrix. The cell e.s.d.'s are taken into account individually in the estimation of e.s.d.'s in distances, angles and torsion angles; correlations between e.s.d.'s in cell parameters are only used when they are defined by crystal symmetry. An approximate (isotropic) treatment of cell e.s.d.'s is used for estimating e.s.d.'s involving l.s. planes.=============================================================================Short contacts:3.1027 (0.0010) Br - O2_$5 2.9569 (0.0014) N3 - O1_$6176.07 (0.05) C4 - Br - O2_$5 117.45 (0.08) Br - O2_$5 - N3_$5 88.60 (0.07) C12 - N3 - O1_$6 133.52 (0.08) N3 - O1_$6 - C8_$6Operators for generating equivalent atoms: $5 *x* - 2, *y* + 1, *z* + 1 $6 - *x* + 1, -*y* + 1, -*z*=============================================================================Least-squares planes (*x*,*y*,*z* in crystal coordinates) and deviations from them (* indicates atom used to define plane)0.3935 (0.0035) *x* + 6.9720 (0.0023) *y* - 6.7731 (0.0052) *z* = 1.9340 (0.0046)* -0.0103 (0.0010) C1 * -0.0015 (0.0010) C2 * 0.0088 (0.0011) C3 * 0.0055 (0.0012) C4 * 0.0052 (0.0011) C5 * 0.0019 (0.0010) C6 * -0.0096 (0.0006) BrRms deviation of fitted atoms = 0.00694.0311 (0.0033) *x* + 7.2756 (0.0034) *y* - 0.4894 (0.0028) *z* = 5.0905 (0.0022)Angle to previous plane (with approximate e.s.d.) = 38.42 (0.04)* 0.0108 (0.0009) C8 * -0.1154 (0.0007) O1 * 0.1064 (0.0009) N2 * 0.0391 (0.0010) C7 * -0.1107 (0.0006) S * 0.0699 (0.0006) N1Rms deviation of fitted atoms = 0.0851- 1.1638 (0.0036) *x* + 5.3585 (0.0037) *y* - 9.2611 (0.0039) *z* = 0.3293 (0.0031)Angle to previous plane (with approximate e.s.d.) = 56.27 (0.04)* -0.0141 (0.0009) C9 * 0.0107 (0.0009) C10 * 0.0018 (0.0009) C11 * -0.0108 (0.0009) C12 * 0.0072 (0.0009) C13 * 0.0053 (0.0009) C14Rms deviation of fitted atoms = 0.00921.5803 (0.0099) *x* - 4.5675 (0.0126) *y* + 10.0071 (0.0113) *z* = 0.2418 (0.0080)Angle to previous plane (with approximate e.s.d.) = 6.52 (0.17)* 0.0000 (0.0000) N3 * 0.0000 (0.0000) O2 * 0.0000 (0.0000) O3Rms deviation of fitted atoms = 0.0000
Refinement. Refinement of *F*^2^ against ALL reflections. The weighted *R*-factor *wR* and goodness of fit *S* are based on *F*^2^, conventional *R*-factors *R* are based on *F*, with *F* set to zero for negative *F*^2^. The threshold expression of *F*^2^ > σ(*F*^2^) is used only for calculating *R*-factors(gt) *etc*. and is not relevant to the choice of reflections for refinement. *R*-factors based on *F*^2^ are statistically about twice as large as those based on *F*, and *R*- factors based on ALL data will be even larger.

### Fractional atomic coordinates and isotropic or equivalent isotropic displacement parameters (Å^2^)

**Table:**

	*x*	*y*	*z*	*U*_iso_*/*U*_eq_	
S	0.23307 (5)	0.59552 (4)	0.59750 (3)	0.01794 (7)	
Br	−0.60756 (2)	1.070560 (16)	0.782568 (13)	0.02754 (5)	
O1	0.14426 (13)	0.61950 (11)	0.23224 (8)	0.0212 (2)	
O2	1.06603 (14)	0.23725 (12)	−0.03589 (9)	0.0267 (2)	
O3	0.84622 (15)	0.09309 (11)	−0.06698 (9)	0.0266 (2)	
N1	0.03832 (16)	0.71824 (12)	0.44897 (10)	0.0159 (2)	
H01	0.015 (2)	0.7119 (19)	0.3861 (13)	0.030 (5)*	
N2	0.31103 (16)	0.56699 (12)	0.37204 (9)	0.0152 (2)	
H02	0.4011 (19)	0.5244 (16)	0.3860 (13)	0.015 (4)*	
N3	0.89393 (17)	0.20238 (13)	−0.02463 (9)	0.0190 (2)	
C1	−0.10821 (18)	0.79834 (14)	0.53147 (11)	0.0151 (3)	
C2	−0.3000 (2)	0.79948 (15)	0.52021 (13)	0.0220 (3)	
H2	−0.3305	0.7441	0.4616	0.026*	
C3	−0.4475 (2)	0.88188 (16)	0.59493 (14)	0.0259 (3)	
H3	−0.5792	0.8833	0.5874	0.031*	
C4	−0.4023 (2)	0.96140 (15)	0.67991 (12)	0.0205 (3)	
C5	−0.2115 (2)	0.96202 (15)	0.69167 (12)	0.0217 (3)	
H5	−0.1817	1.0176	0.7503	0.026*	
C6	−0.0636 (2)	0.88033 (15)	0.61667 (12)	0.0199 (3)	
H6	0.0683	0.8805	0.6237	0.024*	
C7	0.18736 (18)	0.63277 (14)	0.46887 (11)	0.0143 (2)	
C8	0.28512 (19)	0.56083 (14)	0.26223 (11)	0.0159 (3)	
C9	0.44489 (18)	0.47137 (14)	0.18279 (10)	0.0146 (2)	
C10	0.39423 (19)	0.35787 (15)	0.12081 (11)	0.0180 (3)	
H10	0.2593	0.3415	0.1258	0.022*	
C11	0.5409 (2)	0.26914 (15)	0.05200 (11)	0.0181 (3)	
H11	0.5087	0.1905	0.0103	0.022*	
C12	0.73630 (19)	0.29840 (14)	0.04574 (11)	0.0160 (3)	
C13	0.79035 (19)	0.41304 (14)	0.10333 (11)	0.0157 (3)	
H13	0.9256	0.4315	0.0956	0.019*	
C14	0.64264 (19)	0.50033 (14)	0.17260 (11)	0.0154 (3)	
H14	0.6760	0.5798	0.2131	0.018*	

### Atomic displacement parameters (Å^2^)

**Table:**

	*U*^11^	*U*^22^	*U*^33^	*U*^12^	*U*^13^	*U*^23^
S	0.01960 (16)	0.02092 (16)	0.01264 (15)	0.00527 (13)	−0.00323 (12)	−0.00443 (12)
Br	0.02540 (8)	0.02009 (8)	0.02847 (8)	0.00880 (5)	0.00799 (6)	−0.00562 (6)
O1	0.0190 (5)	0.0291 (5)	0.0137 (4)	0.0107 (4)	−0.0025 (4)	−0.0006 (4)
O2	0.0192 (5)	0.0311 (6)	0.0256 (5)	0.0075 (4)	0.0012 (4)	−0.0064 (4)
O3	0.0329 (6)	0.0246 (5)	0.0218 (5)	0.0078 (4)	−0.0055 (4)	−0.0115 (4)
N1	0.0155 (5)	0.0186 (5)	0.0125 (5)	0.0045 (4)	−0.0019 (4)	−0.0041 (4)
N2	0.0139 (5)	0.0178 (5)	0.0127 (5)	0.0066 (4)	−0.0019 (4)	−0.0021 (4)
N3	0.0230 (6)	0.0211 (6)	0.0111 (5)	0.0081 (5)	−0.0022 (4)	−0.0024 (4)
C1	0.0143 (6)	0.0139 (6)	0.0139 (6)	0.0031 (5)	0.0016 (5)	−0.0022 (5)
C2	0.0184 (7)	0.0199 (7)	0.0282 (8)	0.0044 (5)	−0.0060 (6)	−0.0106 (6)
C3	0.0143 (7)	0.0210 (7)	0.0409 (9)	0.0034 (5)	−0.0035 (6)	−0.0112 (6)
C4	0.0198 (7)	0.0134 (6)	0.0216 (7)	0.0039 (5)	0.0066 (5)	−0.0024 (5)
C5	0.0259 (7)	0.0191 (7)	0.0194 (7)	0.0051 (6)	−0.0044 (6)	−0.0066 (5)
C6	0.0159 (6)	0.0217 (7)	0.0220 (7)	0.0033 (5)	−0.0045 (5)	−0.0063 (5)
C7	0.0143 (6)	0.0124 (6)	0.0142 (6)	−0.0009 (5)	0.0004 (5)	−0.0023 (5)
C8	0.0170 (6)	0.0160 (6)	0.0120 (6)	0.0019 (5)	0.0007 (5)	0.0000 (5)
C9	0.0171 (6)	0.0161 (6)	0.0087 (5)	0.0048 (5)	−0.0009 (5)	0.0004 (5)
C10	0.0161 (6)	0.0227 (7)	0.0149 (6)	0.0029 (5)	−0.0040 (5)	−0.0011 (5)
C11	0.0232 (7)	0.0181 (6)	0.0129 (6)	0.0029 (5)	−0.0045 (5)	−0.0036 (5)
C12	0.0185 (6)	0.0170 (6)	0.0099 (5)	0.0073 (5)	−0.0002 (5)	−0.0007 (5)
C13	0.0141 (6)	0.0196 (6)	0.0123 (6)	0.0036 (5)	−0.0019 (5)	−0.0006 (5)
C14	0.0183 (6)	0.0164 (6)	0.0106 (6)	0.0031 (5)	−0.0025 (5)	−0.0017 (5)

### Geometric parameters (Å, °)

**Table:**

S—C7	1.6683 (13)	C9—C14	1.3948 (18)
Br—C4	1.9011 (13)	C9—C10	1.3963 (19)
O1—C8	1.2254 (15)	C10—C11	1.3860 (17)
O2—N3	1.2316 (15)	C11—C12	1.3882 (19)
O3—N3	1.2212 (15)	C12—C13	1.3818 (19)
N1—C7	1.3337 (16)	C13—C14	1.3852 (16)
N1—C1	1.4226 (15)	N1—H01	0.815 (14)
N2—C8	1.3763 (17)	N2—H02	0.774 (12)
N2—C7	1.3938 (15)	C2—H2	0.9500
N3—C12	1.4751 (15)	C3—H3	0.9500
C1—C2	1.3848 (19)	C5—H5	0.9500
C1—C6	1.3898 (19)	C6—H6	0.9500
C2—C3	1.3903 (18)	C10—H10	0.9500
C3—C4	1.377 (2)	C11—H11	0.9500
C4—C5	1.381 (2)	C13—H13	0.9500
C5—C6	1.3898 (18)	C14—H14	0.9500
C8—C9	1.4982 (16)		
			
C7—N1—C1	127.15 (11)	C13—C12—C11	122.97 (11)
C8—N2—C7	128.71 (11)	C13—C12—N3	118.24 (11)
O3—N3—O2	124.19 (11)	C11—C12—N3	118.78 (12)
O3—N3—C12	118.26 (11)	C12—C13—C14	118.42 (12)
O2—N3—C12	117.54 (11)	C13—C14—C9	119.91 (12)
C2—C1—C6	119.98 (12)	C7—N1—H01	116.8 (12)
C2—C1—N1	117.26 (12)	C1—N1—H01	114.9 (12)
C6—C1—N1	122.66 (12)	C8—N2—H02	118.6 (11)
C1—C2—C3	119.77 (13)	C7—N2—H02	112.5 (11)
C4—C3—C2	119.81 (13)	C1—C2—H2	120.1
C3—C4—C5	121.04 (12)	C3—C2—H2	120.1
C3—C4—Br	118.99 (10)	C4—C3—H3	120.1
C5—C4—Br	119.97 (11)	C2—C3—H3	120.1
C4—C5—C6	119.22 (13)	C4—C5—H5	120.4
C5—C6—C1	120.18 (13)	C6—C5—H5	120.4
N1—C7—N2	115.54 (11)	C5—C6—H6	119.9
N1—C7—S	126.05 (10)	C1—C6—H6	119.9
N2—C7—S	118.38 (9)	C11—C10—H10	120.0
O1—C8—N2	123.94 (12)	C9—C10—H10	120.0
O1—C8—C9	122.94 (12)	C10—C11—H11	120.9
N2—C8—C9	113.11 (11)	C12—C11—H11	120.9
C14—C9—C10	120.52 (11)	C12—C13—H13	120.8
C14—C9—C8	119.93 (12)	C14—C13—H13	120.8
C10—C9—C8	119.54 (12)	C13—C14—H14	120.0
C11—C10—C9	119.96 (12)	C9—C14—H14	120.0
C10—C11—C12	118.15 (12)		
			
C7—N1—C1—C2	−140.03 (14)	O1—C8—C9—C14	130.59 (14)
C7—N1—C1—C6	43.7 (2)	N2—C8—C9—C14	−50.80 (16)
C6—C1—C2—C3	−0.5 (2)	O1—C8—C9—C10	−50.40 (18)
N1—C1—C2—C3	−176.90 (13)	N2—C8—C9—C10	128.21 (13)
C1—C2—C3—C4	−0.2 (2)	C14—C9—C10—C11	2.54 (19)
C2—C3—C4—C5	0.6 (2)	C8—C9—C10—C11	−176.47 (11)
C2—C3—C4—Br	−178.99 (11)	C9—C10—C11—C12	−0.99 (19)
C3—C4—C5—C6	−0.3 (2)	C10—C11—C12—C13	−1.09 (19)
Br—C4—C5—C6	179.32 (10)	C10—C11—C12—N3	178.37 (11)
C4—C5—C6—C1	−0.4 (2)	O3—N3—C12—C13	173.49 (11)
C2—C1—C6—C5	0.8 (2)	O2—N3—C12—C13	−5.83 (17)
N1—C1—C6—C5	177.03 (12)	O3—N3—C12—C11	−6.00 (17)
C1—N1—C7—N2	−179.75 (12)	O2—N3—C12—C11	174.68 (12)
C1—N1—C7—S	2.0 (2)	C11—C12—C13—C14	1.60 (19)
C8—N2—C7—N1	−10.14 (19)	N3—C12—C13—C14	−177.87 (11)
C8—N2—C7—S	168.29 (11)	C12—C13—C14—C9	−0.02 (18)
C7—N2—C8—O1	2.3 (2)	C10—C9—C14—C13	−2.02 (19)
C7—N2—C8—C9	−176.27 (12)	C8—C9—C14—C13	176.98 (11)

### Hydrogen-bond geometry (Å, °)

**Table:**

*D*—H···*A*	*D*—H	H···*A*	*D*···*A*	*D*—H···*A*
N1—H01···O1	0.82 (1)	2.03 (2)	2.688 (2)	138 (2)
N2—H02···S^i^	0.77 (1)	2.79 (1)	3.553 (1)	169 (1)
C5—H5···O3^ii^	0.95	2.38	3.293 (2)	162
C3—H3···S^iii^	0.95	2.93	3.474 (2)	118
C14—H14···S^i^	0.95	2.89	3.167 (1)	98
C14—H14···Br^iv^	0.95	3.15	3.899 (1)	137

Symmetry codes: (i) −*x*+1, −*y*+1, −*z*+1; (ii) *x*−1, *y*+1, *z*+1; (iii) *x*−1, *y*, *z*; (iv) −*x*, −*y*+2, −*z*+1.
